# Exploring the Role of Age and Gender on the Impact of Client Suicide in Mental Health Practitioners

**DOI:** 10.1177/00302228221075287

**Published:** 2022-03-01

**Authors:** Edward C. J. Pulleyn, Ruth Van der Hallen

**Affiliations:** 1 Department of Psychology, Education and Child Studies, 6984 Erasmus University Rotterdam, Netherlands

**Keywords:** practitioner, client suicide, demographics, age, gender, short-term impact, IES-R

## Abstract

**Objective:** Previous research has revealed that mental health professionals (MHPs) often experience severe, yet varying, levels of short-term impact in the aftermath of client suicide. Individual differences are significant, yet what factors help explain these differences remains unclear. The current study investigated the role of the MHPs’ and the clients’ age and gender upon the impact of client suicide. **Method:** An international sample of 213 MHPs, aged between 18 and 75, reported on a client’s suicide and its short-term impact (IES-R). **Results:** The results indicate that both MHPs’ and clients’ gender did not affect impact. MHPs’ and clients’ age did not affect impact individually, although a significant interaction effect was revealed. **Conclusion:** Age, not gender, of the MHP and client are relevant in light of the impact of client suicide. Potential implications and suggestions for future research are discussed.

Around the world, suicide—death caused by injuring oneself with the intent to die—is considered a serious global public health problem, despite preventative efforts. According to the World Health Organization (n.d.), nearly 800,000 deaths occur as a result of suicide worldwide every year, amounting to roughly one person every 40 seconds. Mental health disorders such as depression and PTSD are frequently cited as one of several risk factors for suicide; hence, high rates of suicide are found amongst mental health service users. Luoma et al. (2002) revealed that about one-third of suicide victims had contact with mental health services within the last year of their life, and about one-fifth of suicide victims in the month prior to their death. As such, loss due to suicide not only affects friends and relatives but also mental health professionals (MHPs), like psychologists, social workers, counselors, psychiatrists, and psychiatric nurses, who provided care prior. A significant number of MHPs experience a client suicide at some point in their career: in one study, as many as 82% of psychiatrists reported patient suicide, with 47% experiencing the death of a patient within their first 5 years of practice (Cryan et al., 1995). In fact, as a result of the frequency of client suicide within mental health care, it is often referred to as an “occupational hazard” of associated professions (Chemtob et al., 1989).

Research has shown a range of short-term and long-term reactions following client suicide. Over a third of MHPs contemplate a career change following the experience of client suicide due to the resulting stress (Sherba et al., 2019), and negative effects often persist in the long-term, sometimes manifesting in the form of increased anxiety when working with other suicidal clients (Kleespies et al., 1993). However, for the majority of MHPs, the most severe emotional reactions occur shortly after the event (Murphy et al., 2019). One investigation into the immediate consequences of client suicide in psychologists found that those who had experienced the death of a client reported feelings of anger and guilt, in addition to intrusive thoughts regarding suicide, and increased concerns with issues of death (Chemtob et al., 1988). In a qualitative analysis of responses to client suicide, MHPs also reported stress associated with feelings of responsibility for the death, in addition to states of grief and anger (Ting et al., 2006). These complex grief reactions amongst MHPs may sometimes resemble symptoms of post-traumatic stress disorder (Schultz, 2004), highlighting the severity of the impact of client suicide. As such, it is imperative to understand precisely how, why, and when MHPs experience stressful responses to client suicide in order to devise effective targeted support.

Individual differences in the presentation and strength of impact following client suicide are significant (Sandford et al., 2020; Seguin et al., 2014), raising the question of which personal or situational factors may predict impact. Factors that have been investigated concerning the impact following client suicide include the particular role of MHPs in the event (Dransart et al., 2014; Grad et al., 1997; Gulfi et al., 2010; McAdams & Foster, 2000), yet proved to be unrelated. The strength of the clienttherapist relationship (Bowers et al., 2006); greater emotional closeness (Dransart et al., 2014; Gulfi et al., 2010); and longer, more intense involvement with a client (Murphy et al., 2019; Pieters et al., 2003), however, have been identified as (positive) predictors of the impact of client suicide. That said, each of these factors has limited utility in the development and administration of *preventive* measures, as they are not easily or immediately distinguishable, not like, for instance, simple demographic characteristics.

A first demographic characteristic that has been explored in relation to the impact of client suicide is age—several studies have investigated age of the MHP and revealed younger professionals are at greater risk (Chemtob et al., 1988; McAdams & Foster, 2000). Some have theorized that this age effect is linked to a lack of clinical experience, particularly in light of evidence that suggests clinical experience has a protective influence (Draper et al., 2014; Saini et al., 2016). To complicate matters, Murphy et al. (2019) found that older MHPs experience greater personal sadness and grief following client suicide compared to younger MHPs—however, it has been proposed that this effect is a result of recall bias, as older MHPs may have remembered a distressing case of client suicide from much earlier in their career. In other instances, no relationship between the age of MHPs and impact following client suicide has been found (Davidsen, 2011; Gulfi et al., 2010; Hendin et al., 2000; Wurst et al., 2010), adding to the uncertainty. The influence of a client’s age upon impact has received less attention, though several studies have observed that impact is likely greater when the victim of suicide is younger (Dewar et al., 2000; Murphy et al., 2019; Pieters et al., 2003; Wurst et al., 2010).

A second demographic characteristic that has been explored in relation to the impact of client suicide is the gender of the MHP, that is, whether the practitioner in question identifies as male or female. As such, research has revealed a significant effect of gender with greater impact amongst female MHPs (Darden & Rutter, 2011; Draper et al., 2014; Gulfi et al., 2010; Wurst et al., 2013). Moreover, studies have indicated that female MHPs feel greater shame and guilt than male MHPs following client suicide (Grad et al., 1997), and that female MHPs report more intrusive symptoms in the aftermath of client death, whilst male MHPs report more avoidant behaviors (Jacobson et al., 2004). That said, not all scholars have revealed a significant relationship between impact and gender of the MHP (Chemtob et al., 1989; Davidsen, 2011; Dransart et al., 2014; Pilkinton & Etkin, 2003), likely due to differences in the sample (size), making it difficult to draw clear-cut conclusions.

Interestingly, the client’s age and gender or potential interaction between the client and MHP’s age and gender have received no attention in relation to the impact of client suicide. Similarity-attraction theory supposes that individuals tend to like and connect to other individuals who are more similar to themselves (Byrne, 1997). As such, this theory would allow one to expect that characteristic-matching, for instance, a female MHP and female client, would influence the impact following client suicide. In line with that, some previous research has revealed interesting consequences of similarities between MHPs and their clients in general practice. For example, similarities in age between client and practitioner have been found to stimulate growth in therapeutic alliance (Behn et al., 2018) and promote stronger bonds at intake (Rosen et al., 2012). Moreover, similarities in gender between client and practitioner have been linked to stronger therapeutic alliances (Wintersteen et al., 2005), increased attendance of sessions (Lambert, 2016), and a higher satisfaction rate (Johnson & Caldwell, 2011). This in mind, as well as aforementioned research that suggests close therapeutic relationships predict more impact (Bowers et al., 2006; Dransart et al., 2014; Gulfi et al., 2010), it is reasonable to theorize that shared characteristics between client and MHP might also influence impact following client suicide.

The current study aims to conduct an in-depth investigation of the role of clients’ and MHPs’ age and gender upon the short-term impact of client suicide on MHPs. Previous research investigating the role of age and gender in relation to the impact of client suicide has only taken into account age and gender of the MHP, not age or gender of the client, nor the interaction between client and MHP. As such, the current study aims to answer the following research question: How do age and gender of MHPs and their clients influence the impact of client suicide on MHPs? Concerning age, it is hypothesized that age of MHPs, age of clients, and their interaction will have a significant effect on impact, with younger MHPs, younger clients, and similarities in age predicting more impact. Concerning gender, it is hypothesized that gender of MHPs, gender of clients, and their interaction will have a significant effect on impact, with female MHPs, female clients, and similarities in gender predicting more impact. The current study may hold implications for clinical practice, in particular in identifying and supporting at-risk MHPs or in case of at-risk contacts, with demographic variables like age and gender easily identifiable before any therapeutic relationship needs to have been established.

## Method

### Participants

The study included 213 MHP who experienced client suicide at least once prior. Of the 213 MHPs, 53 identified as male (25%), 158 as female (72%), and 2 as non-binary (1%). Age ranged between 18 and 75, with 90% of participants aged between 18–55 years of age. Profession was indicated as psychologist (46%), psychiatric nurse (14%), psychiatrist (13%), counselor (10%), social worker (9%), or other (8%). The majority of the sample originated in Belgium (47%), Germany (18%), and the Netherlands (15%). Of the 213 client suicides MHPs reported on, 113 involved a male client (53%), 99 a female client (46%), and in 1 case a client that identified as non-binary (1%). Client’s aged ranged between −18 and 85+, with 20 clients aged between 25–34 and 45–54 years. Age and gender distributions for MHPs and clients are provided in Table 1.

**Table 1.** Age and gender distributions.

		MHP		Client	
		N	%	N	%
Age	Young (<18 y-34 y)	73	34.3	102	47.9
	Middle-aged (35 y–54 y)	96	45.1	69	32.4
	Older adulthood (55 y–85+)	44	20.7	42	19.7
Gender	Male	53	24.9	113	53.1
	Female	158	74.2	99	46.5
	Non-binary	2	0.9	1	0.5

Note. MHP = mental health professional; N = sample size.

### Procedure

The present study is part of a larger research project looking into the impact of client suicide. Study protocols were in accordance with the ethical standards of the ethical committee of Erasmus University Rotterdam. Participant recruitment was achieved through the online distribution of materials (via email and social media). Individual informed consent was obtained prior to participation. Data was collected using a self-administered, online survey available in English, Dutch, and German. Participants were asked that in case they had experienced multiple client suicide throughout their career, they would answer with their most distressing case of client suicide in mind. Survey completion took approximately 15 minutes.

### Materials

Participants were asked to report on a number of demographics, including their age and gender, and the age and gender of the clients of the respective client suicide. Both age (under 18, 18–24, 25–34, 35–44, 45–54, 55–64, 65–74, 75–84, and 85 or older) and gender (male, female, and non-binary) were collected as categorical variables per request of the ethical committee to ensure the anonymity of both the MHP and the client.

To evaluate the impact of client suicide, the Revised Impact of Event Scale (IES-R; Weiss, 2007) was employed. The IES-R is a revised version of the original IES (Creamer, Bell, & Failla, 2003), a self-report questionnaire developed to assess the impact of a particular (traumatic) event in the 7 days following the event. The IES-R includes 22 items across subscales of intrusion, avoidance, and hyperarousal. Each item is rated on a 5-point Likert scale, with response anchors ranging from 0 “Not at all” to 4 “Extremely.” While the IES-R is not a diagnostic tool for PTSD in itself, the consensus is that sum scores between 24-32 suggest partial PTSD or at least some PTSD symptoms, sum scores between 33-38 suggest a PTSD diagnosis is probable, and sum scores of 39 or above suggest long-term impact (Asukai & Kato, 2002; Creamer et al., 2003). The reliability of the questionnaire was evaluated for the current sample, with α =.95 suggesting excellent reliability.

### Data analysis

To investigate the role of client’s and MHP’s age and gender upon the short-term impact of client suicide on MHPs, two two-way between-measures ANOVAs were conducted using IBM SPSS Statistics 25.0. First, to investigate how age of MHPs and their clients influence the impact of client suicide, a two-way between-measures ANOVA was performed with age of MHP, age of client and their interaction, and independent variables and IES-R sum score as the dependent variable. Second, to investigate how gender of MHPs and their clients influences impact of client suicide, a two-way between-measures ANOVA was performed with gender of MHP, gender of client and their interaction, and independent variables and IES-R sum score as the dependent variable. Age (“young,” middle-aged,” and “older adulthood”) and gender (“male” and “female”) were included as categorical variables, IES-R sum score (0–88) was included as a continuous variable. Before conducting both ANOVA’s, all appropriate statistical assumptions were evaluated. All significance tests were conducted with a significance level of 5%.

## Results

**Figure 1.**
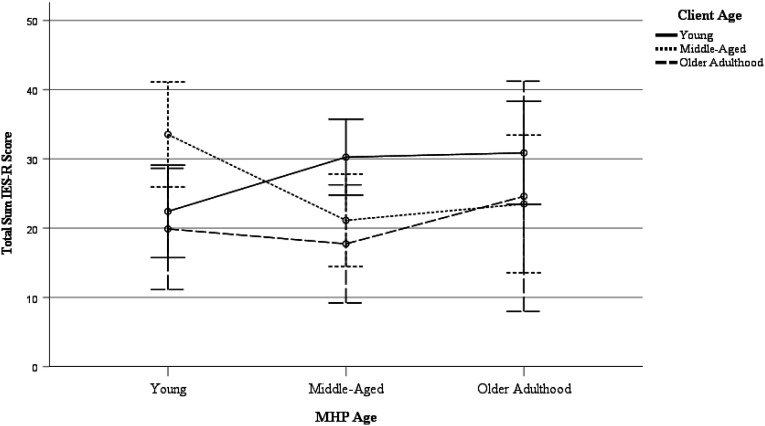
Estimated Marginal Means of IES-R Sum Score. Age was operationalised as Young (<18y -34y), Middle-Aged (35y – 54y), and Older Adulthood (55y – 85+). Error bars represent 95% confidence intervals. MHP = Mental Health Professional.

For descriptive analyses of MHPs’ and clients’ ages and gender in relation to each other, see Table 2. To investigate the role of MHPs’ and clients’ ages upon short-term impact, a two-way between-measures ANOVA was performed, with age of MHPs, age of clients, and their interaction as independent variables, and total sum IES-R score as the dependent variable. Results revealed no main effect of MHP age, *F*(2, 204) = .446, *p* = .641, η^2^ = .004 or client age, *F*(2, 204) = 1.592, *p* = .206, η^2^ = .015, but a significant interaction effect, *F*(4, 201) = 2.549, *p* = .040, η^2^ = .048. Pairwise comparisons revealed significantly more impact for young MHPs in case of middle-aged clients compared to young (*p* = .031) or older clients (*p* = .021) and more impact for middle-aged MHPs for young clients compared to middle-aged (*p* = .038) and older clients (*p* = .016). All other differences were none significant (*p*s > .05). For a visualization of the interaction effect, see Figure 1. Overall, results regarding the age of MHPs and clients suggest that contrary to predictions, the short-term impact of client suicide is not determined by age of MHPs or clients separately, yet, in line with predictions, it is influenced by the interaction between the two.

**Table 2.** Descriptive statistics of MHP and client’s age and gender.

MHP	Client	M	SD	N	%		MHP	Client	M	SD	%
Young	Young	22.42	19.23	31	34		Male	Male	20.36	15.64	25
	Middle	33.54	20.39	24				Female	22.47	18.88	
	Older	19.89	18.79	18							
Middle	Young	30.26	21.81	46	45		Female	Male	25.91	18.86	75
	Middle	21.13	15.73	31				Female	28.23	20.28	
	Older	17.74	11.87	19							
Older	Young	30.88	19.17	25	21						
	Middle	23.50	19.91	14							
	Older	24.60	12.05	5							

Note. MHP = mental health professional; M = mean; SD = standard deviation; N = sample size.

To investigate the role of MHPs’ and clients’ gender upon short-term impact, a two-way between-measures ANOVA was performed, with gender of MHPs, gender of clients, and their interaction as independent variables, and total sum IES-R score as dependent variable. Results indicate no main effect of MHP gender, *F*(1, 206) = 3.282, *p* = .072, η^2^ = .016, client gender, *F*(1, 206) = .503, *p* = .479, η^2^ = .002, nor a significant interaction, *F*(1, 206) = .001, *p* = .974, η^2^ = .000. Overall, these results suggest that contrary to predictions, short-term impact of client suicide is not determined by the gender of the MHPs, clients, or a combination thereof.

## Discussion

The current study aimed to conduct an in-depth investigation of the role of clients’ and MHPs’ age and gender upon the short-term impact of client suicide on MHPs. Concerning age, results revealed no main effect of MHPs’ and clients’ age, yet there was a significant interaction of MHPs’ and clients’ age. More specifically, more impact of client suicide was identified for young MHPs following the suicide of middle-aged clients and middle-aged MHPs following the suicide of young clients. Concerning gender, results revealed no main effect of MHPs’ and clients’ gender nor a significant interaction effect. Potential implications of these findings as well as suggestions for research and clinical practice are discussed next.

With regard to age, results revealed no main effect of MHPs’ and clients’ age, yet there was a significant interaction of MHPs’ and clients’ age. Regarding the age of MHPs, some previous studies have revealed an effect of age on the impact of client suicide, with either older MHPs (Murphy et al., 2019) or younger MHPs (Chemtob et al., 1988; McAdams & Foster, 2000) reporting more impact following client suicide. Yet, most previous work has failed to find such an effect (Davidsen, 2011; Gulfi et al., 2010; Hendin et al., 2000; Wurst et al., 2010) in line with the current results, suggesting there is no inherent generational difference. Moreover, the current study also did not reveal an effect of client’s age, while most previous work has identified a relationship between a client’s age and the impact of client suicide (Dewar et al., 2000; Murphy et al., 2019; Pieters et al., 2003; Wurst et al., 2010), with more impact in case of younger clients. The most plausible explanation here is that the current study was the first to take the interaction between MHPs’ and clients’ age into account, shining a different light on previously revealed main effects of age.

Interestingly, the interaction between MHPs’ and clients’ age within the current study proved to be significant, with more impact of client suicide for young MHPs following the suicide of middle-aged clients and for middle-aged MHPs following the suicide of young clients. No previous research has investigated such an interaction effect. Surprisingly, no similarity effect was revealed—that is, situations where the MHP and the client were of the same age were not associated with more impact following client suicide. One possible explanation is that the current effects are, instead, the result of more parental-like countertransference dynamics. Natural evolutionary tendencies toward caregiving exist in parents and nonparents alike (Schaller, 2018), and the loss of a client to suicide could usher mental comparisons similar to the loss of a parental figure or child, increasing impact following client suicide as a result. Remarkably, a similar effect was not present for any of the MHP age categories in relation to older clients. Previous research has shown that the passing of older adults is generally correlated with an increased preparedness and a reduced shock in their relatives (Barry et al., 2002). Furthermore, some of these instances may have involved so-called “rational suicides”, that is, sane, well-thought-out decisions formulated by mentally competent and capable individuals choosing the best alternative among the many available without ambivalence (Gramaglia et al., 2019). Consider, for instance, older or elderly clients that express suicidal wishes in the absence of overt mental or significant physical illness. MHPs are increasingly encountering rational suicide (and witness to coinciding changes in the socio-political climate related to euthanasia, physician-assisted death, and physician-assisted suicide), which may, in turn, affect their view of elderly suicide altogether (Balasubramaniam, 2018; Gramaglia et al., 2019). All things considered, further investigation is required to test these abovementioned suggestions. Irrespective of the cause of these age-related differences, these findings may hold implications for support strategies in mental health care. The current results implicate that while the age of the MHP or the client cannot be used as such to identify at-risk clinical contact, the importance of a relative difference in age, as revealed by the interaction effect, can prove valuable in identifying at-risk clinical contact or subsequent loss.

With regard to gender, the current results revealed no effect of MHPs’ or clients’ gender nor a significant interaction effect between the two. Most previous research has identified a significant relationship between the gender of the MHP and the impact of client suicide, with female MHPs reporting more impact (Darden & Rutter, 2011; Draper et al., 2014; Gulfi et al., 2010; Wurst et al., 2013). That said, in line with current results, a significant number of scholars have not revealed such a relationship either (Chemtob et al., 1989; Davidsen, 2011; Dransart et al., 2014; Pilkinton & Etkin, 2003). The persistent disparity between the outcomes of conducted studies is intriguing, to say the least. As the current mental health workforce is predominantly female (Lin et al., 2018), it is reassuring that female MHPs are not consistently more impacted by client suicide, yet it does not explain why some studies have observed gender differences and others have not. One possible explanation is that there are no gender differences, beyond the fact that female MHPs may be more inclined to open up in self-report questionnaires. Previous research suggests that males tend to underreport depressive symptoms (Sigmon et al., 2005), and it is not unlikely that tendency might be true for the emotional impact of trauma as well. Alternatively, researchers may find it easier to interpret or publish data when it aligns with the common (mis)conception that females are more emotional (Barrett et al., 1998). Ultimately, the current results imply that in terms of allocating support in light of the impact of client suicide, one should not distinguish between male and female MHPs (though the type of support that is desired might still differ).

Interestingly, no previous studies have investigated the role of the client’s gender nor the interaction between the MHPs’ and the clients’ gender. The current study revealed neither the client’s gender nor the interaction between the gender of the MHP and their client are particularly important with regard to the impact of client suicide. While male suicides are more common compared to female suicides (Callanan & Davis, 2012), male versus female client suicides do not elicit a different response in MHPs. Likewise to what was revealed for age, no similarity effect was revealed for gender, meaning whether a client and a MHP are of the same gender or not, was not associated with impact following client suicide. That said, as previous research has found significant effects of gender similarity upon variables like therapeutic alliance (Wintersteen et al., 2005) and therapy attendance (Lambert, 2016), it should not be discounted that some type of similarity effect of gender might still exist; for instance, it is possible that such an effect is strongest during early days of contact with a client and simply dissipates over time when other factors, like the therapeutic relationship, come to play a bigger role. Taken together, the current results with regard to gender suggest that neither an MHP’s gender nor a client’s gender nor the relationship between the two needs particular consideration when trying to identify at-risk clinical contact or potential subsequent loss. Rather than focusing on MHPs’ and client’s age and gender, the current result suggests other moderators of the effect, like the strength of the client-therapist relationship (Bowers et al., 2006), duration of treatment (Murphy et al., 2019; Pieters et al., 2003), previous exposure to suicide (Van der Hallen, 2021), coping styles (Darden & Rutter, 2011; S. Sanders et al., 2008), and attitudes toward suicide (Boukouvalas et al., 2020; Kodaka et al., 2013), should be considered when designing effective pre- or postvention initiatives. This is of particular importance as MHPs’ risk of mental illness and suicide is significantly increased (Milner et al., 2013; Dutheil et al., 2019) and current pre- and postvention initiatives, in particular those related to suicidality, are not typically offered in postgraduate training programs (Berlim et al., 2007; Oordt et al., 2009).

Three limitations should be considered when interpreting or generalizing the current results. First, it is probable that the current study does not take cultural differences into account properly and so the extent to which these findings may be generalized worldwide should be reviewed cautiously. Notably, the current dataset is an international sample, yet collected from primarily Western nationalities. It is not unlikely that these results would differ in societies that hold more varying attitudes toward individuals of different genders or ages. For example, Japan is renowned for a strong sense of respect and obligation towards the elderly (Koyano, 1989), whereas in Western societies, older individuals are customarily seen as less socially valuable (Sanders et al., 1984). Secondly, the current study opted to use the IES-R (Weiss, 2007), a self-report tool commonly used to assess the impact of a particular (traumatic) event, in this case, a client suicide. Although the IES-R is a preferred tool among studies investigating trauma, there are inherent issues of reliability with the use of self-report scales, such as that self-reported data may be affected by social desirability bias, embarrassment, or a failure to remember details correctly (Stone et al., 1999). The latter issue is of particular concern as MHPs are asked to recall an instance of client suicide that may have occurred years ago—at worst, such response biases could skew data or obscure the presence of significant effects. Corroboration of information from co-workers, friends, and family who engaged with participants immediately following client suicide could increase the reliability of data, as would the collection of data sooner to the occurrence of each incident. Last but not least, the employed sample suffered from uneven group sizes. Although the overall sample size was large and the chosen analyses are robust against differences in group size, the dataset did contain a greater proportion of female compared to male MHPs. Similarly, sample sizes for both MHPs and clients in older adulthood were limited. Banding age categories or re-analyzing datasets with more balanced distributions of participants across groups may result in somewhat different results.

To conclude, the current study conducted an in-depth investigation of the role of clients’ and MHPs’ age and gender upon the short-term impact of client suicide on MHPs. Results revealed no effect of the age of the MHP or the client, nor the gender of the MHP or the client, yet there was an interaction between the age of the MHP and age of the client. Overall, the current results suggest age and gender are only of minimal importance when deciding how to allocate or provide support in light of the impact of client suicide in MHPs. Future research may seek to investigate these effects further, in particular, the interaction between MHP and client demographics, with more comprehensive samples, additional measurement scales of impact, or additional variables like the strength of the therapeutic relationship.
